# Potential Fast COVID-19 Containment With Trehalose

**DOI:** 10.3389/fimmu.2020.01623

**Published:** 2020-07-07

**Authors:** Daisy Martinon, Vanessa F. Borges, Angela C. Gomez, Kenichi Shimada

**Affiliations:** ^1^Division of Infectious Diseases and Immunology, Department of Pediatrics, Cedars-Sinai Medical Center, Los Angeles, CA, United States; ^2^Department of Biomedical Sciences, Cedars-Sinai Medical Center, Los Angeles, CA, United States

**Keywords:** trehalose, SARS-CoV-2, COVID-19, prophylaxis, autophagy

## Abstract

Countries worldwide have confirmed a staggering number of COVID-19 cases, and it is now clear that no country is immune to the SARS-CoV-2 infection. Resource-poor countries with weaker health systems are struggling with epidemics of their own and are now in a more uncertain situation with this rapidly spreading infection. Frontline healthcare workers are succumbing to the infection in their efforts to save lives. There is an urgency to develop treatments for COVID-19, yet there is limited clinical data on the efficacy of potential drug treatments. Countries worldwide implemented a stay-at-home order to “flatten the curve” and relieve the pressure on the health system, but it is uncertain how this will unfold after the economy reopens. Trehalose, a natural glucose disaccharide, is known to impair viral function through the autophagy system. Here, we propose trehalose as a potential preventative treatment for SARS-CoV-2 infection and transmission.

## Introduction

The pandemic coronavirus disease 2019 (COVID-19), caused by severe acute respiratory syndrome coronavirus 2 (SARS-CoV-2), has rapidly expanded around the globe. As of June 5, 2020, the World Health Organization (WHO) has confirmed 6,515,796 cases and reported 387,298 deaths worldwide due to COVID-19. The currently available treatments aim to control symptoms, guarantee tissue perfusion, and avoid multiple organ dysfunction syndrome. This is accomplished through the use of oxygen therapy, mechanical ventilation in respiratory failure cases, and hemodynamic support for managing septic shock (1). Unfortunately, advanced healthcare systems are struggling to keep up with the influx of COVID-19 patients in need of treatment and hospitalization. An increasing concern is the detrimental impact this virus could have in countries with weaker health systems and vulnerable populations. At this time, there is no known U.S. Food and Drug Administration (FDA)-approved treatment that can effectively control SARS-CoV-2 infection and transmission. This review aims to propose trehalose as a prospective prophylactic treatment for COVID-19 containment.

Trehalose (α,α-trehalose), a naturally occurring sugar, is a non-reducing disaccharide with two glucose molecules that functions as an energy source in many organisms. Trehalose is present in various organisms, such as plants, algae, fungi, yeast, bacteria, insects, and other invertebrates. In 2000, the U.S. FDA gave trehalose a generally recognized as safe (GRAS) status (2). Because of its protectant effects against freezing and dehydration, trehalose has become an attractive ingredient in food, health, and pharmaceutical products (2). The optimized production process of trehalose makes it accessible and affordable. Although humans cannot synthesize trehalose, the small intestine can hydrolyze trehalose into two D-glucose molecules by the specific enzyme trehalase. Since the hydrolyzing activity of trehalase is ordinarily low, it causes only a mild increase in blood glucose and insulin levels (3, 4). In contrast, sucrose, a disaccharide composed of glucose and fructose, immediately increases both blood glucose and insulin levels after oral intake (5). On the other hand, unhydrolyzed trehalose can be rapidly absorbed in the bloodstream after oral administration to mice, rats, and macaques, which peaked at ~15 min to 1 h post-dose and continued to be detectable in macaques up to 12 h post-dose (6, 7). The pharmacokinetic data on non-human primates provide a promising incentive for human response to trehalose. In human subjects, the daily intake of 10 g/day trehalose for 12 weeks improved glucose tolerance, decreased the progression to insulin resistance, and decreased systolic blood pressure. Therefore, trehalose can induce systemic benefits that are not associated with an increase in glucose levels and insulin resistance (8). Altogether, these studies support the feasibility and safety of trehalose treatment for the prevention of SARS-CoV-2 infection and transmission.

Since 1925, several studies aimed to understand the chemical and biological properties of trehalose. Trehalose has received attention for its protective properties in neurodegenerative and metabolic diseases (9–12). The protective effects of trehalose were considered to be due to its chemical chaperone properties (13). However, in 2007, trehalose was identified as an autophagy inducer (14). Autophagy, a highly conserved intracellular degradation process, is regulated by the major control complex mammalian target of rapamycin (mTOR) (15, 16). Interestingly, trehalose induces autophagy independent of mTOR (14). In mammalian cell cultures, trehalose treatment can enhance the clearance of denatured huntingtin and denatured α-synuclein, both autophagy substrates associated with Huntington's and Parkinson's diseases, respectively (14). The trehalose effects are dependent on the accumulation of microtubule-associated protein 1A/1B-light chain 3 (LC3) II, which is essential for the formation of autophagosomes from autophagy-related 5 (ATG5) protein complex anchored phagophores (14). However, these are independent of mTOR inhibition, since trehalose does not reduce the phosphorylation levels at specific sites on mTOR substrates, which are reduced by the mTOR inhibitor rapamycin (14). Later, the transcription factor EB (TFEB) was identified to be the primary lysosomal biogenesis regulator, as it induces the expression of several essential genes for this phenomenon (17). When trehalose emerged as an autophagy inducer, some research groups were compelled to use trehalose to demonstrate the role of TFEB. It was observed that trehalose reduced the activity of AKT (protein kinase B), which phosphorylates TFEB (S467A), thus enabling the translocation of TFEB to the nucleus independently of the TFEB inhibitor, mTOR (18). Furthermore, trehalose can activate TFEB and could subsequently promote cross-presentation (18, 19).

In maternal diabetes, repressed autophagy in developing neuroepithelium cells leads to neural tube defects (NTDs) in the developing fetus (20). Trehalose administration restored this autophagy defect and prevented the hyperglycemia-induced NTDs (21). In another *in vitro* study, trehalose promoted LC3 autophagosome formation in high glucose cultured cells, higher clearance of ubiquitin-binding protein p62, neural stem cell differentiation, mitophagy, and reticulophagy (22). Sanfilippo syndrome or mucopolysaccharidosis (MPS) type III is a neurodegenerative lysosomal storage disorder. A mouse model of MSP III, treated with trehalose, showed neurological improvements associated with the clearance of autophagic vacuoles in neuronal and glial cells, as well as the activation of the TFEB transcription network (23). In a model of myocardial infarction cardiac remodeling, the use of trehalose resulted in an improved outcome in wild-type mice (24). On the other hand, the trehalose administration to mice genetically deficient in Beclin-1, an important protein in autophagy regulation, had no protection (24). Trehalose also increased LC3 and p62 clearance. Conjointly, these results confirmed that trehalose-induced autophagy elicited cardioprotective effects (24). Trehalose administration also inhibited and attenuated high-fat diet-induced atherosclerosis in mice and rabbits, respectively (25–27). Mashhad University of Medical Sciences is now sponsoring an interventional clinical trial to test the beneficial effects of intravenous administration of trehalose (15 g per week for 12 weeks) as an anti-inflammatory agent against vascular inflammation and atherosclerosis (clinicalTrials.gov, NCT 03700424). Peripheral arterial disease (PAD) patients are associated with vascular complications and increased platelet activation. Reduced glucose oxidation, oxidative stress, and autophagy are factors that contribute to reduced blood flow in PAD patients (28). A clinical trial approaching the effects of trehalose on endothelial function, oxidative stress, platelet function, and autophagy in PAD patients is currently active (clinicalTrials.gov, NCT 04061070).

**Figure 1:**
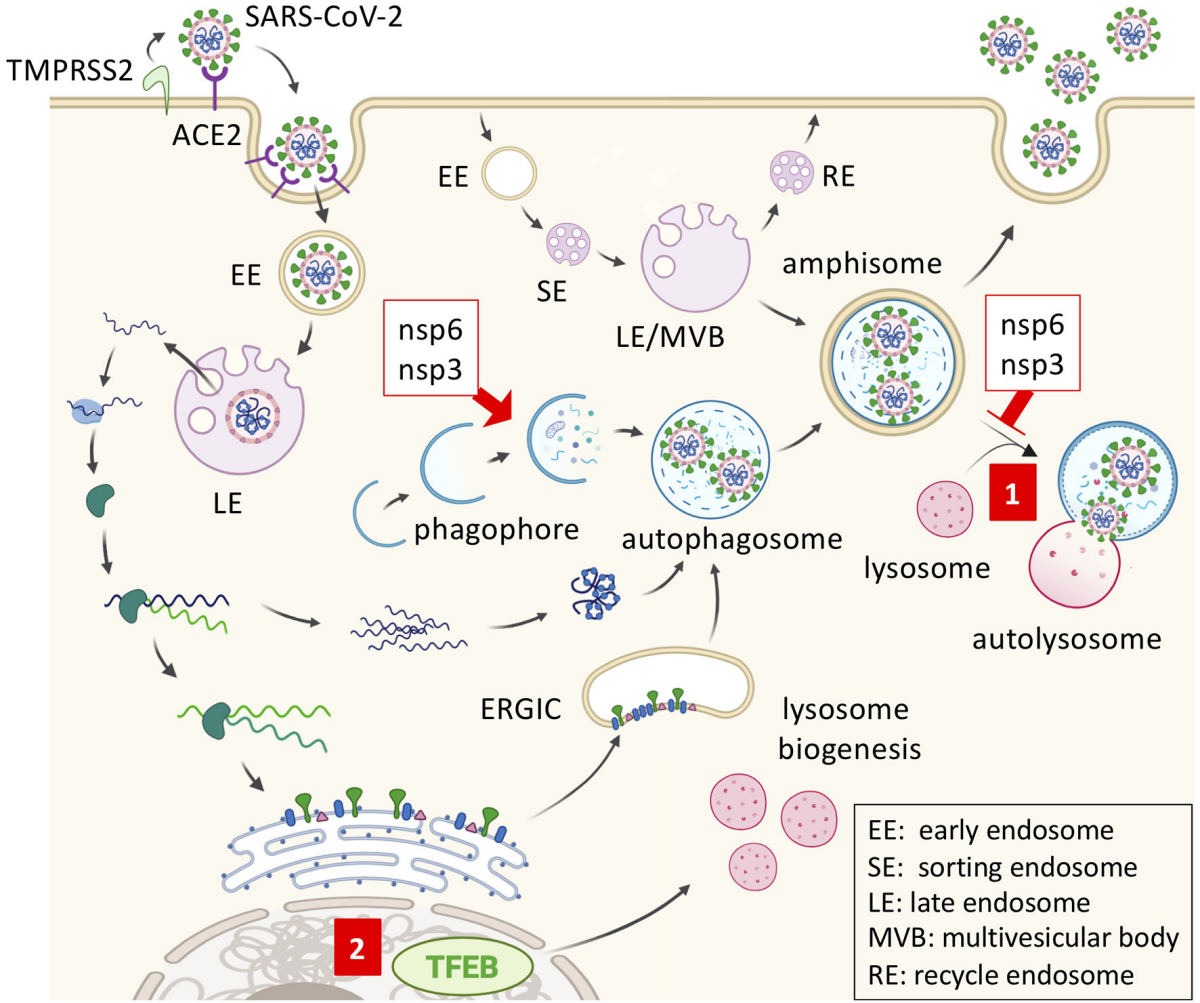
SARS-CoV2 replication and inhibition by trehalose. SARS-CoV-2 gains entry via membrane fusion by binding angiotensin-converting enzyme 2 (ACE2) in the target cell. The virus is then internalized and transported through endosomal compartments. The viral genome is released and used by the host ribosome to synthesize viral RNA polymerase, thus replicating the viral RNA subgenomes. Viral structural proteins are synthesized and anchored into the endoplasmic reticulum (ER). Once the virion is assembled in the ER-Golgi intermediate compartment (ERGIC) and transferred to the autophagosome, it may be released by exocytosis from the amphisome. Coronavirus nonstructural protein 6 (nsp6) and nsp3 promote autophagosome formation but prevent autophagosome-lysosome fusion. (1) Trehalose may promote amphisome-lysosome fusion for virion degradation after the trafficking of viral double-membrane vesicles. (2) Trehalose can also activate transcription factor EB (TFEB) and promote lysosome biogenesis.

In the context of infectious diseases, autophagy induction is critical for both innate and adaptative immune response development. Although some microorganisms can evade autophagy and even use it as a survival and propagation strategy, autophagy is an effective way to control most of intracellular infections (29). The human immunodeficiency virus (HIV), the pathogen well-known for evading immune responses, is also known for its effects in the autophagy flux (30). HIV increases the number of autophagosomes and impairs autophagosome maturation, thus blocking autophagosome-lysosome fusion (31). Despite the fact that CD4^+^ T lymphocytes are the predominant target for HIV infection, it is the HIV-infected innate immune cells that make organisms highly susceptible to opportunistic microorganisms, such as non-pathogenic mycobacteria (32). Although autophagic inhibition by 3MA or Beclin-1 siRNA decreased HIV virus production in the Jurkat cell line (31), a recent study has shown that trehalose can restore autophagy impaired by HIV infection in peripheral blood mononuclear cells (PBMCs) *in vitro* (Table 1) (32). In addition, trehalose post-treatment reduced HIV load in PBMCs from HIV infected patients (32). Antiviral effects of trehalose on other disease-related viruses, such as human cytomegalovirus (HCMV) and varicella-zoster virus (VZV), have been demonstrated *in vitro* (Table 1). Its mechanism of action lies in its ability to induce autophagy significantly. Trehalose inhibited HCMV viral gene expression and viral spread in multiple cell types (34). Both pre- and post-treatments with trehalose reduced HCMV and VZV replication (35). Interestingly, Clark et al. (2018) reported in HCMV infected cells that trehalose alters the intra-multivesicular bodies (MVBs) virion morphology and redirects the trafficking of viral vacuole maturation through downregulation of Ras-like GTPase 11 (Rab11), a protein involved in the control of endosomal trafficking (Figure 1) (33). In primary mouse cortical cultures infected with the West Nile virus (WNV), trehalose induced robust activation of autophagy indicated by an increase in protein expression of LC3-II; however, activation of autophagy had no significant effect on WNV growth (37) (Table 1). Similar autophagy activation was observed in human primary airway epithelial cells treated with trehalose, but autophagy induction impaired antiviral function against human rhinovirus (HRV) infection (36) (Table 1).

**Table 1:** Summary of antiviral effects by trehalose.

**Virus**	**Main findings/observations**	**Effective dose**	**References**
Human immunodeficiency virus (HIV)	Trehalose post-treatment reduced HIV-1 in PBMCs from healthy donor Trehalose *ex vivo* treatment reduced HIV-1 in HIV patient PBMCs	100 mM	(32)
Herpes cytomegalovirus (HCMV)	Trehalose altered intra-MVB virion morphology Trehalose did not change viral DNA synthesis Trehalose increased lysosomes Trehalose disrupted Rab11, altered virus trafficking	50, 100 mM	(33)
Herpes cytomegalovirus (HCMV)	Trehalose increased autophagosome in HCMV infected cells Trehalose inhibits HCMV gene expression and virus production in HFFs, HAECs and in neuronal culture	50, 100 mM	(34)
Herpes cytomegalovirus (HCMV)	Pre- and post-treatment of trehalose reduced anti-HCMV infected cells	50, 100 mM	(35)
Human rhinovirus (HRV)	Trehalose increase LC3-II in HRV infected human primary airway epithelial cells Trehalose reduced IFNi-1 mRNA, and promoted HRV replication	100 mM	(36)
West Nile virus (WNV)	Viral titer was not changed *in vitro* at low dose trehalose	10 mM	(37)
Varicella-zoster virus (VZV)	Pre- and post-treatment of trehalose reduced anti-VZV in infected cells	100 mM	(35)

The beta-coronaviruses, severe acute respiratory syndrome coronavirus (SARS-CoV) and Middle East respiratory syndrome coronavirus (MERS-CoV), encode viral proteins that impair autophagosome-lysosome fusion, such as the membrane-associated papain-like protease (PLp-TM) (38). Also, coronaviruses replicate in LC3-mediated autophagosome-like double-membrane vesicles in an ATG7-independent manner (39, 40). Since various viruses hijack the autophagic machinery to the benefit of viral replication and virion maturation, regulating cellular autophagy with autophagy modulators such as chloroquine (CQ) or trehalose is of great importance in the treatment of viral diseases (41). In regard to this, coronaviruses express non-structural protein 6 (nsp6) and nsp3 that promote autophagosome formation but prevent autophagosome-lysosome fusion, indicating that autophagy flux induction by trehalose could be beneficial for potential COVID-19 containment (Figure 1) (38, 42). The angiotensin-converting enzyme 2 (ACE2) plays an essential role as a SARS-CoVs receptor to mediate viral entry through endocytosis (Figure 1) (43). Of note, the recently described LC3-associated endocytosis (LANDO), that occurs with the conjugation of LC3 to Rab5^+^ clathrin^+^ endosomes containing β-amyloid, is another newly reported important mechanism to clear the exogenous antigens in microglial cells (44). LANDO may be another underestimated LC3-vesicle with the potential for coronaviral replication, which can explain the LC3-dependent and ATG7-independent coronaviral replication observed previously. However, the dichotomy lies in that both LC3-phagosome and LC3-endosome are single-membrane vesicles, while coronaviral replication would occur in double-membrane vesicles (40). So far, there is no known effect of trehalose on LANDO, yet it is clear that trehalose displays autophagy-dependent antiviral activities against different types of viruses.

## COVID-19 Therapeutic Strategies, Developments and Challenges

The major strategies to slow down the spread of COVID-19, thus reducing the strenuous demands on healthcare, were to isolate those infected in their household and promote social distancing (45, 46). However, in light of the continuously increasing cases, “stay-at-home” is the measure that many countries are adhering to in order to suppress the spread. New COVID-19 cases are accelerating every minute, and experts do not know how long it will take before this pandemic is under control. Meanwhile, the loss of commerce, trade, tourism, and global supply chains has economic experts grappling on the detrimental effects this virus will have on the world economy for years to come. The global economy predicted a $280 billion loss in the first quarter of the year without urgent global action (47). The United Nations Conference on Trade and Development (UNCTAD) predicts a preliminary $2 trillion shortfall in the global economy (48).

In response to the COVID-19 pandemic, the U.S. FDA continues its work to ensure safe and effective treatments. Potential therapies that are being investigated and developed for the treatment and management of COVID-19 now comprise of numerous antiviral agents, immunotherapies, and vaccines (49). The antiviral nucleotide analog agent remdesivir can inhibit the replication of SARS-CoV and MERS-CoV in multiple *in vitro* systems, such as primary human airway epithelial cell cultures (50, 51). Remdesivir has shown promising results in rhesus macaques infected with SARS-CoV-2, and the Adaptive COVID-19 Treatment Trial for remdesivir, sponsored by the National Institute of Allergy and Infectious Disease, has been completed (clinicalTrials.gov, NCT 04280705) (52). Based on this trial and the Gilead-sponsored trial (clinicalTrials.gov, NCT 04292899), the U.S. FDA has authorized emergency use of remdesivir for the treatment of COVID-19 (53). Lopinavir/Ritonavir, an HIV protease inhibitor, was found to inhibit SARS-CoV-2 replication in Vero E6 cells (54). Hospitalized COVID-19 patients in Wuhan, China, were treated with the combinational therapy of lopinavir/ritonavir. However, the effect was moderate, and 13% of the patients had to be removed early in the treatment due to adverse side effects (55). New triple antiviral therapy combination of interferon beta-1b, lopinavir-ritonavir, and ribavirin in the treatment of mild to moderate COVID-19 patients was safe and superior to lopinavir-ritonavir treatment alone in alleviating symptoms, shortening the duration of viral shedding, and hospital stay. However, the lack of a placebo group was one of several limitations to this trial (56).

CQ and its structural analog hydroxychloroquine (HCQ), FDA approved drugs used as antimalarial agents with anti-inflammatory and immunomodulatory activities, are another investigational antiviral therapy drugs being redirected and evaluated for COVID-19 treatment. *In vitro* studies have shown a significant activity of CQ against SARS-CoV-2, SARS-CoV-1, and MERS-CoV (49, 57). CQ is a lysosomotropic agent and inhibits both endosomal and autophagosomal acidification, thereby, impairing the functionality of fused endosomes-lysosomes as well as fused autophagosome-lysosome (autolysosome) (58–60). CQ inhibits coronaviral RNA entry into the cytosol, which is required for viral replication after ACE2-mediated endocytosis (Figure 1) (61). Pre-clinical rationale and evidence moved CQ into the clinical trial phase for the treatment of COVID-19. The FDA approved the emergency use of CQ and HCQ for COVID-19 patients (62). Unfortunately, there is still no large randomized trial that proves these drugs to be effective against the disease. Interestingly, azithromycin and erythromycin, macrolide antibiotics, have specific antiviral effects that can inhibit viral internalization. Oseltamivir-azithromycin combination therapy has been used in randomized clinical trials for the treatment of influenza (63). The French Ministry of Health approved an HCQ-azithromycin open-labeled non-randomized clinical trial on SARS-CoV-2 infected patients and showed promising results in the small sample size study (64). Unfortunately, it has been well-documented that arrhythmias are a potential risk in COVID-19 patients treated with CQ-azithromycin (65, 66).

Remdesivir, lopinavir/ritonavir, CQ, and azithromycin have shown to be effective in inhibiting SARS-CoV-2 replication *in vitro*. However, the efficacy diminishes when symptomatic patients are treated 1 week after the disease's onset. At this point, the patient may have already transitioned from viral expansion to the inflammatory phase leading to a cytokine storm (67). For instance, during influenza infection, the viral shedding phase peaks 2–3 days after infection (68). A meta-analysis of randomized controlled trials of oseltamivir (Tamiflu) indicates that early treatment of the infection accelerated clinical symptom alleviation and reduced the risks of lower respiratory tract complications, suggesting that antiviral drug treatments have to be timed appropriately (69, 70). Antiviral drug monotherapy might not be sufficient for severe patients with COVID-19, and supplementation with anti-inflammatory drugs would be necessary as a combination therapy (71). Sanofi and Regeneron have announced a Phase 2/3 trial of interleukin-6 (IL-6) inhibitor sarilumab (Kevzara) to ameliorate tissue damage in the lung caused by a “cytokine storm” in patients with COVID-19 infection (clinicalTrials.gov, NCT 04315298) and Swedish Orphan Biovitrum (SOBI) also started Anakinra (IL-1 receptor antagonist) and Emapalumab (Anti-IFN-γ monoclonal antibody) Phase 2/3 trials to test their efficacy to reduce hyper-inflammation and respiratory distress in patients with COVID-19 infection (clinicalTrials.gov, NCT 04324021). Overall, there are more clinical research trials to be made before any of these drugs become available to the public.

Efforts are also underway for the development of vaccines against SARS-CoV-2. Major COVID-19 vaccines under evaluation are whole virus vaccines (adenovirus-vectored vaccine using AdVac and PER.C6 technology by Janssen), recombinant protein subunit vaccines (recombinant nanoparticle technology by Novavax), and nucleic acid vaccines (mRNA vaccine by Moderna/NIH/CEPI) (72). A vaccine developed in a pandemic paradigm will take months to several years to carry from a developmental stage to commercialization (72–74). Therefore, a faster and more affordable approach to COVID-19 containment is necessary. Because of its antiviral properties, availability, and FDA approval, trehalose merits immediate evaluation as a promising preventative treatment for COVID-19.

- Several non-specific antiviral drugs are undergoing clinical trials in only a few designated hospitals. While the SARS-CoV-2 viral shedding timeline is not well-known, antiviral drugs would be more beneficial if administered during the onset of the disease. More accurate and faster diagnostic tests are still underway, as a timely diagnosis is key to the management of COVID-19 (75).
- There are no medications or clear guidelines for asymptomatic or mildly symptomatic patients that test positive (76, 77). These patients must remain isolated at home, which increases the chances of infecting other family members.
- Socio-economic disadvantaged countries with low-quality medical health care systems will be at a disadvantage compared to the U.S. and some European countries that are conducting the latest strategies against COVID-19 (78, 79). New low-cost strategies are desperately needed.
- Vaccine development has started in several countries, but this would take at least 1 year to be available.
- Asymptomatic patients may be receiving false-negative PCR test results, which causes underestimation of COVID-19 positive cases. Thus, it complicates efforts to prevent the spread of the virus within the community (80).
- Current lockdown measures could limit the spread, but may not be sufficient to stop it completely.

## Perspective on Trehalose Treatment for Immediate SARS-CoV-2 Containment

To date, the immediate interventions for SARS-CoV-2 transmission have centered on contact tracing, quarantine, and social distancing. Adaptation of these physical distancing measures to mitigate the pandemic has managed to flatten the curve in some countries (81). Cities worldwide have begun to reopen their economy; however, a second wave of COVID-19 cases and deaths is predicted to begin 2–4 weeks after reopening (82). Post-pandemic transmission dynamics of SARS-CoV-2 project recurrent outbreaks during the wintertime and resurgence of this disease may persist for another 3 years (83). Mathematical modeling of various scenarios with one-time social distancing measures has simulated a resurgence of infection when social distancing measures were lifted (83). Thus, additional methods that circumnavigate enforced social distancing and shutting down the economy are needed. Alternatively, the pathways to herd immunity will require either mass vaccination or natural immunization over time. However, the latter approach suggests that a large portion of the population would succumb to the disease (84). The R_0_, basic reproductive number, represents the transmissibility of infectious agents in a naive population and can vary depending on transmission dynamics (85). As such, the R_0_ differs across populations. The R_0_ of SARS-CoV-2 has been reported to range from 1.4 to 6.49 in recent studies from January 2020 – February 2020 (86). With an R_0_ of 6.49, the population would require 85% of individuals to have immunity to abate the spread of disease, 1 – 1/R_0_. It is uncertain how long it will take for different populations to acquire herd immunity due to factors of transmission dynamics, such as demography, cultural influences, contact rates, and population density (87). Therefore, targeting individuals who are considered low-risk could build community protection to protect those that are vulnerable and not immune.

**Figure 2:**
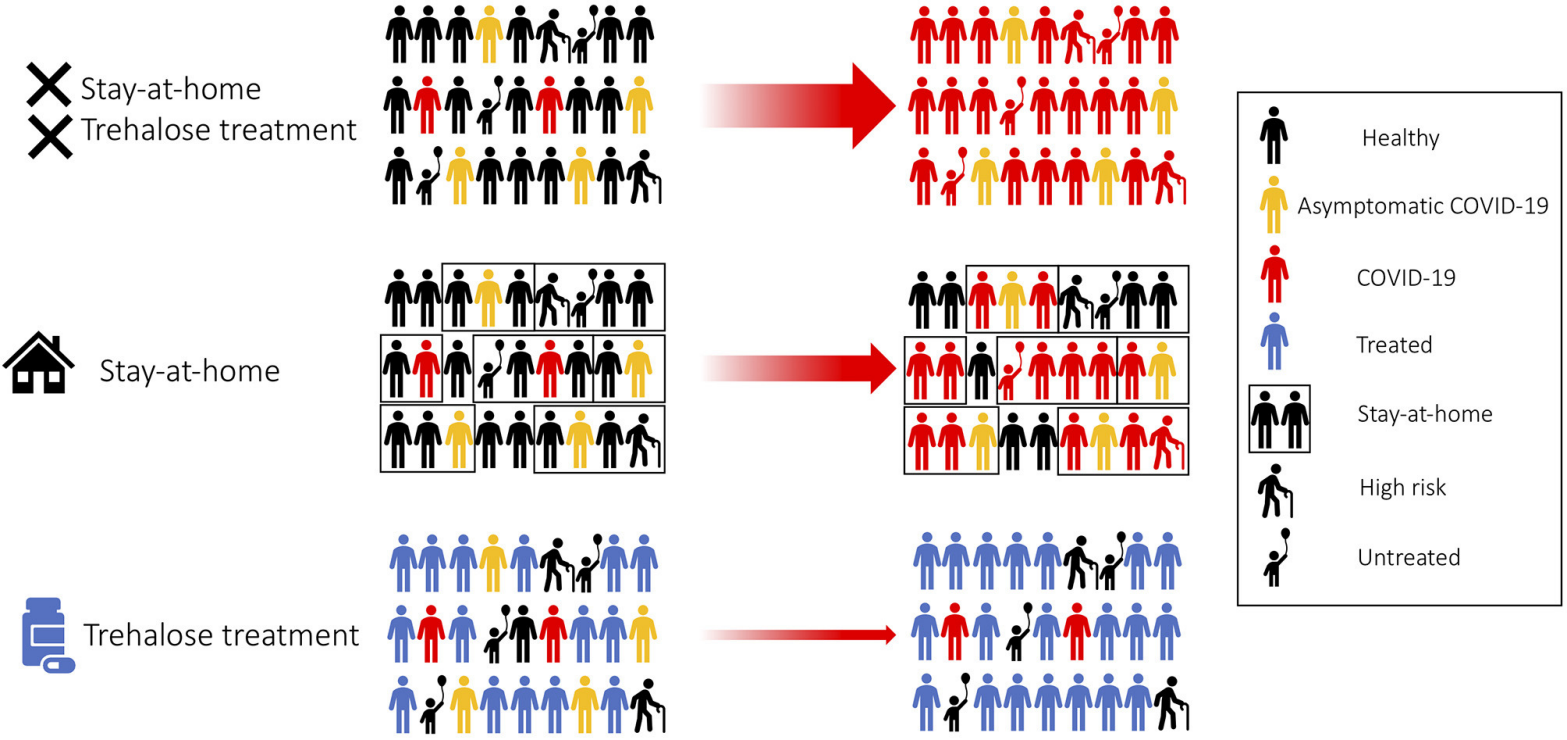
Trehalose treatment for fast SARS-CoV-2 containment. Treating healthy adults and asymptomatic patients with trehalose would accelerate community viral containment.

We propose trehalose as a practical and affordable safe treatment for the prevention and containment of COVID-19. Trehalose could potentially affect cellular organelles required for viral replication. We would recommend a large community trial comprising of healthy adults, healthcare workers, asymptomatic, and mild symptomatic COVID-19 patients (Figure 2). The Centers for Disease Control (CDC) has recently reported the transmission of SARS-CoV-2 during the presymptomatic stage of the infection; therefore, trehalose treatment as a prophylactic approach would be advisable (88, 89). Family members of patients testing positive for COVID-19 will be recommended to undergo trehalose treatment and stay at home in self-quarantine, as the secondary attack rate of SARS-CoV-2 transmission within a household is 16.3% (Figure 2) (90). Unlike adults, children have a low observed case rate of COVID-19. Children are mostly asymptomatic, or the symptoms are mild to require medical attention unless an underlying health-compromising condition exists (91, 92). More recently, pediatric hospitals are confirming cases of children suffering from a severe Kawasaki-like disease related to COVID-19 (93). This is a significant concern when countries are trying to reopen their education system, and the long term effects of SARS-CoV-2 infection are still unknown in children. Clinical studies on the oral supplementation of trehalose (100 g/day) for the improvement of vascular function has been carried out in healthy adults aged 50–77 years (94). Although trehalose has proven to be safe, it would be inappropriate or approached with caution when evaluating the efficacy of trehalose in seniors and the elderly, as a large number of this population suffer from chronic diseases. By treating healthy adults with trehalose, population-level resistance to the infection will block the chain of transmission. As a result of this treatment, the risk of transmission to seniors and medically compromised individuals will be drastically reduced as they are indirectly protected from the infection (Figure 2) (95). Furthermore, trehalose can activate TFEB and could subsequently promote cross-presentation (18, 19). Therefore, trehalose could have another benefit to accelerate herd immunity against SARS-CoV-2, especially in the phase of encountering a second wave of COVID-19 cases. We believe that this treatment strategy can be more beneficial to countries that do not have the resources to conduct antiviral clinical trials.

## Potential Precautions and the Current Limitations for the Development of Trehalose Treatment

It is important to understand that while there is considerable research on trehalose administration in adults, there is still not sufficient data on its safety in children, adults with health-related issues, and the elderly. We have previously reported that trehalose exacerbated acute respiratory distress syndrome (ARDS) “two-hit” murine model (induced by LPS and mechanical ventilation), while other autophagy activations inhibited ARDS (96). Therefore, trehalose treatment would be inappropriate to treat intensive care patients with severe COVID-19, especially those presenting cytokine storm syndrome or ARDS. For inflammatory cytokines, trehalose has been shown to suppress LPS-induced IL-1β and TNF-α secretion in mouse peritoneal macrophages (97). Our experimental *in vitro* data shows that trehalose pretreatment inhibited LPS induced IL-6 production in macrophages (unpublished). As in the clinical practice guidelines by the infectious diseases society of America on the management of influenza, early treatment with antiviral medications attenuates symptoms and risk complications. In the same way, trehalose could have a more significant benefit as an anti-inflammatory in the initial phase of the SARS-CoV-2 infection. Treating severe COVID-19 patients in the late stage of the disease would not be sufficient. Instead, other emergency FDA approved pharmaceutical interventions would be more effective. While trehalose could provide significant benefits as an antiviral treatment, studies have shown that dietary trehalose may enhance the virulence of *Clostridium difficile* (98). *In vitro* studies on the effects of trehalose on SARS-CoV-2 infection still need to be investigated since some type of viruses can highjack the autophagy system and enhance their replication with trehalose treatment (e.g., HRV) (36). Once confirming the effectivity of trehalose, tests in the rhesus macaque model would be ideal for establishing an appropriate starting dose for human clinical trials. The majority of trehalose administration in adults ranges from 4.8 to 10 g/day. Lung alteration of autophagy has been confirmed with the use of 1 g/kg trehalose to mice, which is equivalent to 4.8 g/day for every 60 kgs in humans (96). A diabetes study used 10 g/day (8), while an acute coronary artery clinical trial used 2.1 g/day (clinicalTrials.gov, NCT 03700424). The dose of 30 g has been used for laxative purposes in healthy females (3). An effective dosage of trehalose per body weight for targeting SARS-CoV-2 and pharmacokinetics of trehalose in human blood remains to be determined. Of note, fasting between 16 and 18 h is also known to activate autophagy; however, it is not a good strategy for viral infection as previously reported (99). Thus, we do not recommend fasting as a prophylactic treatment for COVID-19. Rapamycin, a representative mTOR-dependent autophagy activator, has an immunosuppressive adverse effect that may make it inadequate as a prophylactic treatment of SARS-CoV-2 (15).

As SARS-CoV-2 continues to expand globally, it is crucial to gain knowledge and understanding of its impact on disparate communities. The proposed trehalose treatment could potentially protect those unable to seek more costly therapeutics. With COVID-19 studies and trials ongoing indefinitely and economies suffering, more cost-efficient options should be considered for fast containment. The investigation of safer treatments for viral infections should be a continuous priority, as the COVID-19 pandemic will not be the last of its kind. We suggest trehalose for consideration to public health agencies worldwide.

## Author Contributions

DM, VB, AG, and KS made substantial contributions to the conception, writing, and editing of this review.

## Conflict of Interest

The authors declare that the research was conducted in the absence of any commercial or financial relationships that could be construed as a potential conflict of interest.
